# Factors Affecting the Patient’s Decision When Selecting Their Dermatologist in Kuwait: A Cross-Sectional Study

**DOI:** 10.7759/cureus.88835

**Published:** 2025-07-27

**Authors:** Ali Alroumi, Hasan Ashkanani, Nourah Al Ajmi, Zainab Al Mousa

**Affiliations:** 1 Dermatopathology, As'ad K. Al-Hamad Dermatological Center, Kuwait City, KWT; 2 Dermatology, Amiri Hospital, Kuwait City, KWT; 3 Community Medicine, Kuwait University, Kuwait City, KWT

**Keywords:** dermatologist, dermatology, kuwait, patient preferences, selection factors

## Abstract

Background

Dermatology in Kuwait has rapidly expanded to include both medical and cosmetic services. Despite this growth, there has been sparse research attention on what influences patients when selecting dermatologists. This study examines modifiable and non-modifiable factors affecting patient choices.

Objective

This study aims to assess the relative importance of modifiable and non-modifiable characteristics in patients’ selection of dermatologists in Kuwait and to compare these preferences across different demographic and socioeconomic groups.

Method

A cross-sectional online survey with 43 questions was distributed to individuals aged 18 and above in Kuwait. Adapted from a validated tool, the survey assessed preferences related to dermatologist traits. A total of 992 (98.6%) responses were analyzed using non-parametric tests due to non-normal data distribution. Group comparisons were made based on gender, income, governorate, and insurance status.

Results

Professionalism was rated the most important factor (median=10), followed by availability and appearance (median=9). Religion, marital status, and nationality were least important (median=1-2). Female respondents placed greater emphasis on aesthetics, communication, and skincare. Higher-income participants prioritized qualifications like foreign training and research. Regional variation was noted: southern governorates valued appearance, while Jahra favored clinical experience. Insurance status had minimal effect, although uninsured individuals valued appearance and professionalism more.

Conclusions

Patients in Kuwait prioritize modifiable traits - especially professionalism, experience, and accessibility - when choosing dermatologists. Non-modifiable factors play a minor role. While demographic and socioeconomic factors influence preferences to some extent, professionalism remains a consistently valued trait across all groups.

## Introduction

Physician selection may be one of the most essential steps any individual in need of medical attention may take, and it often determines the effectiveness of patient care and satisfaction. This is particularly the case in dermatology, which often involves multiple visits to the treating physician, including an initial consultation and follow-up assessments. In the past few years, the field has grown to encompass numerous cases with varying severities and requirements, ranging from simple pharmacological interventions to even surgical procedures. Patients have also gone to rely on dermatologists for different reasons, including both medical complaints and cosmetic concerns [1]. Nonetheless, patients may still further evaluate dermatologists by factors beyond clinical competence, such as appearance, personality, and social presence [2,3]. Studies have shown that such characteristics, including age, gender, and nationality can affect a patient’s trust and satisfaction, which is a pattern seen in specialties involving long-term care [4-6].

In Kuwait, dermatology is a rapidly growing profession, with increased demand for both therapeutic and aesthetic services. However, very little research has been done to explore the decision-making process patients undergo in selecting their dermatologist [7]. This study investigates the modifiable and non-modifiable factors that influence an individual’s preferences when choosing their dermatologist in Kuwait: non-modifiable factors include but are not limited to a dermatologist’s age, gender, and nationality, whereas modifiable factors include but are not limited to a dermatologist’s experience, professionalism, and presence. Understanding patients’ selection processes and preferences may help dermatologists locally to tailor their practices and better meet expectations and improve satisfaction [8,9].

## Materials and methods

Methodology

An online questionnaire consisting of 43 questions was used and created using Google Forms (Microsoft, Redmond, WA), with a few questions based on a questionnaire done by Bornstein et al. (2000) [8]. Although adapted from it, the modified survey was not subjected to formal psychometric validation after cultural adaptation and translation. The study focused on patient preferences for physician characteristics, like age, gender, nationality, and character - having participants rank them on a scale from 0 to 10, with 0 being not important at all and 10 being most important. A total of 13 items were adapted: doctor’s age, gender, race, religion, attire, reputation, certification, years of experience, medical school, participation in continuing medical education, office waiting time, location, and friendliness. However, several culturally relevant questions were added to the original tool based on a focused literature review and consultation with two board-certified dermatologists familiar with local patient attitudes. The adapted questionnaire was then translated into Arabic using a forward-backward translation method. Two independent bilingual medical professionals conducted the forward translation, followed by a backward translation by a third bilingual researcher. The final Arabic version was reviewed by the research team to ensure conceptual equivalence and clarity. Ethical approval was obtained from the Permanent Committee for Coordination of Medical and Health Research, Ministry of Health, Kuwait (Approval Number: 109/9/2024; approved on November 18, 2024). The survey was distributed through various social media platforms, including WhatsApp, Instagram, and X (formerly Twitter) from December 1, 2024 to January 1, 2025. Everyone had access to an informed consent form before continuing to participate in the survey, which clearly stated that participation was voluntary, responses would be kept confidential, and participants had the right to withdraw at any time without consequence. Individuals aged 18 years and above residing in Kuwait who completed the online questionnaire in full were included in the study, whereas those under 18 years, not residing in Kuwait, or partially completed surveys were excluded from analysis.

Statistical analysis

Data was analyzed using IBM SPSS Statistics version 27 (IBM Corp., Armonk, NY). Normality was assessed using Shapiro-Wilk and Kolmogorov-Smirnov tests. Non-parametric tests like the Mann-Whitney U and Kruskal-Wallis tests were used due to non-normal distribution of most variables. Descriptive statistics (medians and interquartile ranges) and inferential statistics (p-values) were reported. Significance was set at p<0.05.

## Results

**Figure 1:**
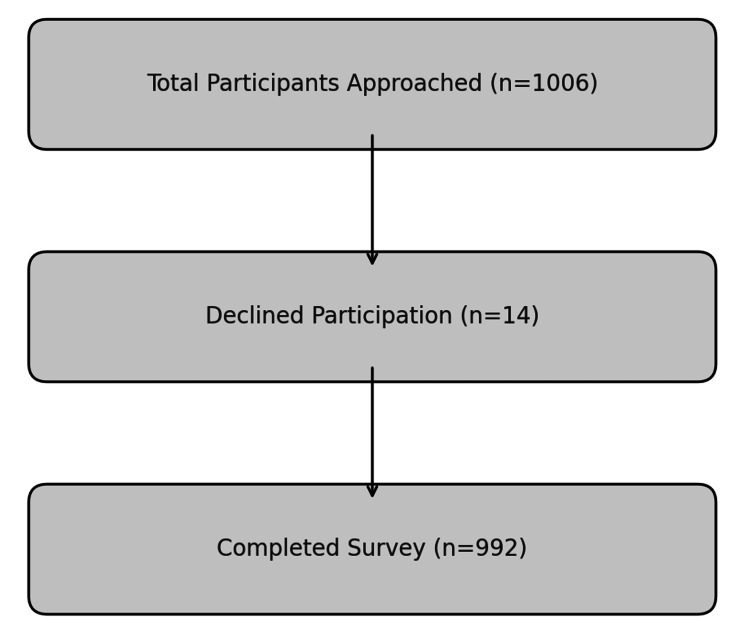
Study Flow Chart

The study was distributed to 1006 participants aged 18 and above in Kuwait, of whom 992 (98.6%) had completed it (Figure 1). The sample’s median age was 33 years and had an interquartile range of 21, with the minimum being 18 years old and the maximum being 84 years old. The age variable was non-normally distributed and was confirmed as such by the Shapiro-Wilk (p<0.001) and Kolmogorov-Smirnov (p<0.001) tests, necessitating the use of non-parametric tests (Mann-Whitney U and Kruskal-Wallis) for subsequent age-related analyses. 

Of all participants who completed the questionnaire, 644 (64.9%) were women and 348 (35.1%) were men. Most participants were Kuwaiti (950; 95.8%), followed by non-Kuwaiti Arabs (38; 3.8%) and non-Arab non-Kuwaitis (4; 0.4%). In terms of educational levels, 636 (64.1%) held a bachelor’s degree or diploma, 250 (25.2%) had completed postgraduate studies (Master’s/Ph.D.), 102 (10.3%) completed high school or secondary education, and only four (0.4%) had less than a high school education. Most were employed (616; 62.1%), with 240 (24.2%) being unemployed and 136 (13.7%) being retired. Income levels were distributed as follows: 240 participants (24.2%) reported earning less than 1000 Kuwaiti Dinar (KD) per month, 262 (26.4%) earned between 1001 and 1500 KD, 194 (19.6%) earned between 1501 and 2000 KD, and 296 (29.8%) earned more than 2000 KD monthly. When asked about health insurance status, 410 participants (41.3%) had health insurance, while 582 (58.7%) did not. As for clinic visits, 320 participants (32.3%) reported no dermatologist visits in the past year, 552 (55.6%) visited a dermatologist one to five times, 86 (8.7%) reported 6-10 visits, and 34 (3.4%) had more than 10 visits.

Comparisons across demographic groups were evaluated using chi-square tests for categorical variables, and Mann-Whitney U or Kruskal-Wallis tests for continuous, non-normally distributed variables such as age. Significance was set at p<0.05, and full test statistics are reported in the analytical sections that follow (Table 1).

**Table 1:** Characteristics of the Study Participants in Kuwait (N=992) KD: Kuwaiti Dinar.

Demographic Variable	N (%)
Nationality	
Kuwaiti	950 (95.8%)
Non-Kuwaiti (Arab)	38 (3.8%)
Non-Kuwaiti (Non-Arab)	4 (0.4%)
Gender	
Male	348 (35.1%)
Female	644 (64.9%)
Education Level	
Less than High School	4 (0.4%)
High School / Secondary	102 (10.3%)
Bachelor’s / Diploma	636 (64.1%)
Higher than Bachelor’s	250 (25.2%)
Employment Status	
Employed	616 (62.1%)
Unemployed	240 (24.2%)
Retired	136 (13.7%)
Marital Status	
Single	524 (52.8%)
Married	402 (40.5%)
Divorced	42 (4.2%)
Widowed	24 (2.4%)
Income Level	
Less than 1000 KD	240 (24.2%)
1001-1500 KD	262 (26.4%)
1501-2000 KD	194 (19.6%)
More than 2000 KD	296 (29.8%)
Health Insurance	
Yes	410 (41.3%)
No	582 (58.7%)
Dermatologist Visits (per year)	
0	320 (32.3%)
1-5 times	552 (55.6%)
6-10 times	86 (8.7%)
More than 10 times	34 (3.4%)
Age (years)	
18-35	534 (53.8%)
36-64	392 (39.5%)
>65	52 (5.2%)

Patient preferences in dermatologist selection

**Figure 2:**
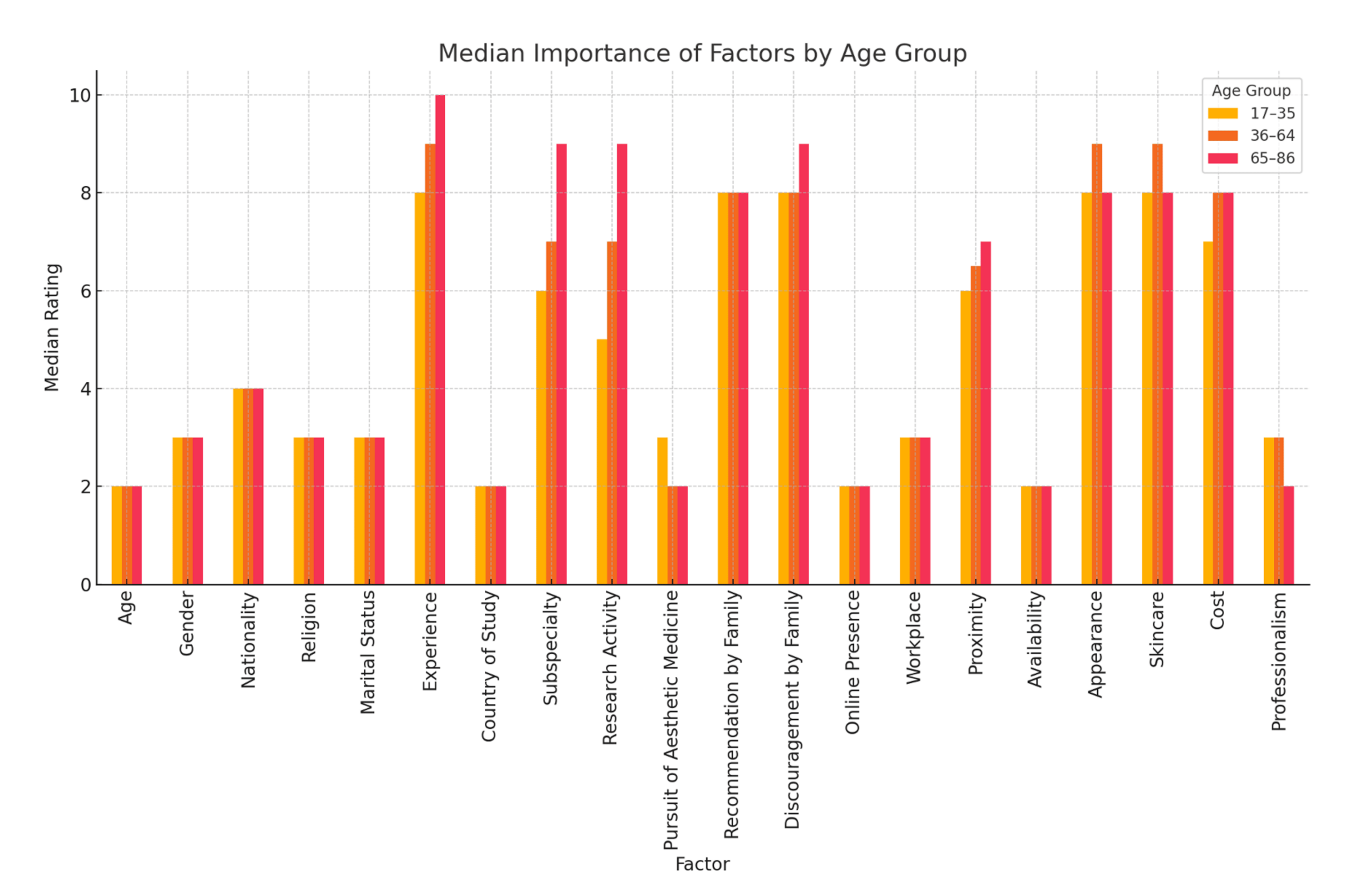
Medians of Each Factor in Dermatologist Selection According to Age Groups

Participants in the study evaluated 20 factors influencing their choice of dermatologist using a 10-point Likert scale. Due to the non-normal distribution of most items, medians and interquartile ranges were used. Group comparisons were analyzed using Mann-Whitney U tests for two-group comparisons, and Kruskal-Wallis tests for variables with more than two categories (Figure 2).

Of all attributes, professionalism was most important to patients (median=10, interquartile range (IQR)=2), followed by both physical appearance and availability (median=9, IQR=3), then followed by experience (median=8, IQR=2). Other factors, such as the physician’s skincare and cost of care were also rated highly (median=8). On the other hand, religion and marital status were the least important when considering which dermatologist to choose (median=1), followed by nationality (median=2) and gender (median=3).

Certain questions further evaluated the specifics of patients’ preferences (Table 2). In terms of a dermatologist’s age, 403 (40.3%) of participants favored middle-aged dermatologists (40-60 years), while 272 (27.4%) preferred those younger than 40 years, and only eight (0.8%) preferred dermatologists older than 60; 315 (31.5%) had no preference. In terms of gender, 550 participants (55.4%) reported no preference, while 297 (29.9%) preferred women and 145 (14.6%) preferred men. This suggests that most patients are flexible, but a notable portion still value gender alignment, particularly toward female providers. With regards to nationality, 610 (61.5%) had no preference, while 337 (34%) preferred Kuwaiti dermatologists. And with regards of religion, 750 (75.6%) reported no preference, although 242 (24.4%) preferred Muslim dermatologists. Similarly, marital status was unimportant to 954 (96.2%) of respondents. These findings indicate that non-modifiable traits play relatively minimal roles in patient decision-making.

**Table 2:** Categorized Preferences

Question (Q#)	Response Option	N (%)	p-value (difference across variable categories; Chi-square)
Q12 - Preferred Age	<40	272 (27.4%)	0.034
	40–60	403 (40.3%)	
	>60	8 (0.8%)	
	No preference	315 (31.5%)	
Q13 - Preferred Gender	Male	145 (14.6%)	0.021
	Female	297 (29.9%)	
	No preference	550 (55.4%)	
Q15 - Preferred Nationality	Kuwaiti	337 (34.0%)	0.049
	Non-Kuwaiti	45 (4.5%)	
	No preference	610 (61.5%)	
Q17 - Preferred Religion	Muslim	242 (24.4%)	0.001
	Non-Muslim	0 (0.0%)	
	No preference	750 (75.6%)	
Q19 - Preferred Marital Status	Single	13 (1.3%)	0.000
	Married	25 (2.5%)	
	No preference	954 (96.2%)	
Q22 - Country of Study	Kuwait	83 (8.4%)	0.028
	Abroad	441 (44.5%)	
	No preference	468 (47.2%)	
Q26 - Aesthetic Pursuit	Highly favorable	219 (22.1%)	0.015
	Slightly favorable	217 (21.9%)	
	Unfavorable	252 (25.4%)	
	No preference	304 (30.6%)	
Q30 - Online Presence	Highly favorable	324 (32.7%)	0.033
	Slightly favorable	344 (34.7%)	
	Unfavorable	115 (11.6%)	
	No preference	209 (21.1%)	
Q32 - Workplace	Private	93 (9.4%)	0.022
	Public	98 (9.9%)	
	Both	534 (53.8%)	
	No preference	267 (26.9%)	
Q34 - Availability	No waiting	289 (29.1%)	0.019
	Short waiting	444 (44.8%)	
	Long waiting	48 (4.8%)	
	No preference	211 (21.3%)	
Q39 - Professionalism vs. Friendliness	Professionalism	393 (39.6%)	0.011
	Friendliness	63 (6.3%)	
	Both equally	536 (54.0%)	

Other questions revolving around modifiable attributes were asked. For country of study, 468 (47.2%) expressed no preference, 441 (44.5%) preferred foreign-trained dermatologists, and 83 (8.4%) favored those trained in Kuwait. Workplace affiliation also showed variance. When it came to workplace, 534 (53.8%) preferred dermatologists working in both private and public sectors, while 267 (26.9%) had no preference. Availability was important to most, with 444 (44.8%) and 289 (29.1%) preferring short or no wait times, respectively. Likewise, online presence was favorable for 668 (67.4%) of respondents, with 324 (32.7%) perceiving it as being highly favorable, while 209 (21.1%) had no preference. For pursuit of aesthetic medicine, responses were mixed: 219 (22.1%) perceived dermatologists with such services as being highly favorable and 217 (21.9%) saw it as being slightly favorable, whereas 252 (25.4%) perceived such activities as being unfavorable, and 304 (30.6%) had no preference. Finally, professionalism stood out: 536 (54.0%) viewed professionalism and friendliness as being equally important, while 393 (39.6%) preferred professionalism over friendliness, and only 63 (6.3%) prioritized friendliness alone. This indicates that patients expect a balanced professional demeanor but place slightly greater weight on professionalism in clinical interactions. These findings suggest that while some preference factors are universally prioritized, others - especially those related to credentials, online presence, and character - vary significantly by demographic background and socioeconomic status.

Gender-based differences

**Figure 3:**
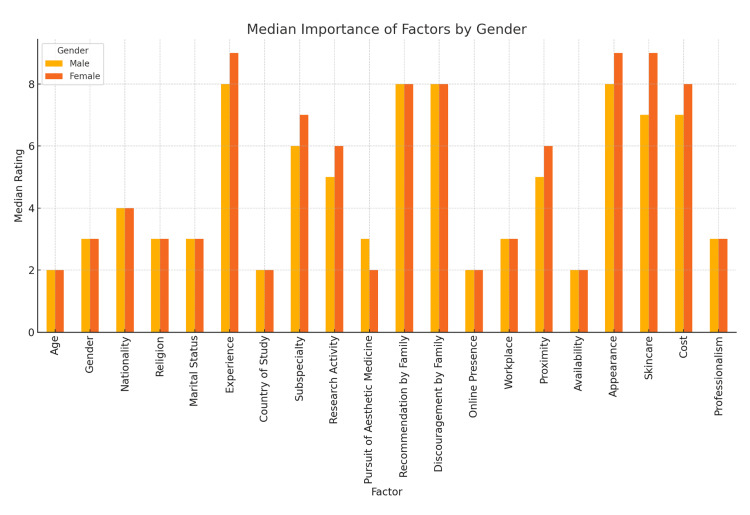
Gender-Based Comparison

When analyzing gender-based differences in dermatologist selection preferences, several notable patterns emerged (Figure 3). Women participants consistently rated more factors as important compared to men, particularly those related to aesthetics, professionalism, and patient-centered care. For instance, professionalism had a median of 10.0 for both genders, but women rated it significantly higher in mean (9.15 vs. 8.43), with a narrower interquartile range (IQR=1 vs. 2). This difference was statistically significant (p=0.001). Similarly, experience was rated higher by women (median=9.0, IQR=3) than men (median=8.0, IQR=4), with a significant p-value (p=0.003). Aesthetic-related perceptions also showed strong gender variation. Appearance was rated higher by women (median=9.0) than men (median=8.0), and the dermatologist’s personal skincare was significantly more important to women (median=9.0, IQR=2) compared to men (median=7.0, IQR=4), with a p-value<0.001. This suggests that women are more influenced by how well dermatologists care for their own skin, potentially viewing it as a reflection of how their own outcome might be. Social influence also differed by gender: recommendation by family and discouragement by family had similar medians for both groups (8.0), but women had higher means and tighter IQRs, indicating a stronger consensus; both were statistically significant (p=0.01 and p=0.016, respectively).

In contrast, factors such as religion and marital status had very low medians (1.0) and did not differ significantly between genders (both p>0.05), suggesting a shared disinterest in these characteristics. Gender of the dermatologist, however, did vary: women had a median of 4.0, while men had a median of 1.0, with p=0.031, reflecting stronger preference for gender concordance among female patients.

Overall, women demonstrated statistically stronger preferences across a range of professional, aesthetic, and interpersonal traits, while men exhibited more neutral patterns. These findings emphasize the value of gender-sensitive approaches in the field.

Socioeconomic differences

**Figure 4:**
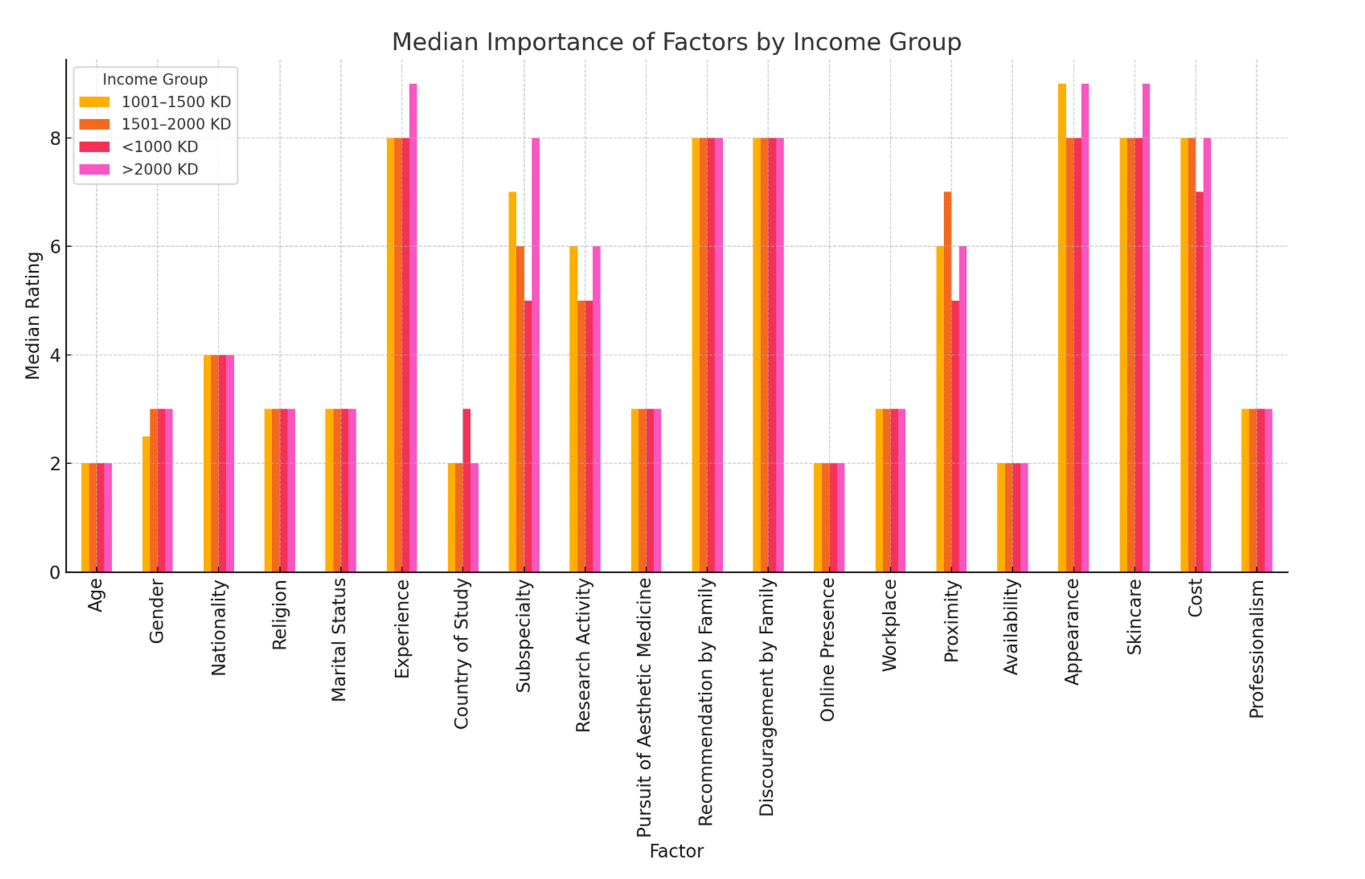
Income-Based Comparison

Income level had a marked impact on patients’ prioritization of factors when selecting a dermatologist (Figure 4). While certain factors like professionalism and experience were highly rated across all income brackets (median=10 and 8-9, respectively), higher-income individuals consistently placed greater importance on professional and academic credentials. For example, a dermatologist’s country of study showed increasing median values with income - from 6.0 in the <1000 KD group to 8.0 in the >2000 KD group - accompanied by a wider interquartile range (IQR=5), reflecting greater diversity of opinion. Similarly, subspecialty and research activity had higher median scores among respondents with higher income (medians=8 and 6, respectively), suggesting that such patients may be more sensitive about academic background and specialized expertise. These differences were statistically significant (p<0.05). Furthermore, aesthetic and image-based factors were also rated higher by wealthier participants. Appearance and personal skincare of a dermatologist both had a median of 9.0 in the >2000 KD group versus 8.0 and 7.5 in the 1001-1500 KD and <1000 KD groups, respectively. Cost, on the other hand, did not follow a linear trend; although all groups rated it as important (median=7-8), the variation across income levels was small and not statistically significant.

Conversely, sociocultural factors such as religion, marital status, and nationality had uniformly low medians across all income brackets (mostly median=1-2), and showed no meaningful difference by income level (p>0.05), indicating that such demographic characteristics are minimally relevant regardless of socioeconomic status. These findings suggest that higher-income patients value academia, professional image, and expertise more strongly than those with lower incomes. The results also reveal that patients across all income groups agree on the universal importance of professionalism and experience and largely disregard non-medical demographic traits.

Residential differences

Analysis across the six governorates of Kuwait - Assimah, Hawally, Jahra, Farwanya, Mubarak Al-Kabeer, and Ahmadi - revealed subtle but noteworthy variations in patient preferences for dermatologist selection (Table 3), with adjustments to other variables. Overall, most questions showed consistent medians, indicating shared core values among participants regardless of location. For instance, professionalism was ranked extremely high across all regions, with a median of 10.0 in all six governorates and minimal interquartile ranges, reinforcing it as a universally prioritized factor. However, some distinctions emerged. Dermatologist’s experience showed a slightly higher median of 10.0 in Jahra compared to 8.0-9.0 in other areas, and country of study had higher medians in Ahmadi and Mubarak Al-Kabeer (median=8.0) versus 6.0-7.0 elsewhere, suggesting that patients in southern governorates may value academic pedigree more strongly. Similarly, physical appearance and skincare tended to score higher in Mubarak Al-Kabeer (median=9.0-10.0), highlighting regional emphasis on the dermatologist's personal presentation.

**Table 3:** Governorate-Based Comparison

Question	Assimah (Median)	Hawally (Median)	Ahmadi (Median)	Farwanyah (Median)	Mubarak Al Kabeer (Median)	Jahra (Median)	p-value
Age	5	5	5	5	6	5	0.224
Gender	3	4	1.5	4	1	4	0.031
Nationality	1	3	1	3	3	3.5	0.028
Religion	1	1	1	1	1	1	0.518
Marital Status	1	1	1	1	1	1	0.477
Experience	8	8	9	9	9	10	0.043
Country of Study	6	6	8	6	8	6	0.017
Subspeciality	7	6	7	8	7	7	0.054
Research Activity	6	6	6	6	6	6	0.662
Pursuit of Aesthetic Medicine	5	5	5	5	5	3	0.045
Recommendation by Family	8	8	8	8	8	8	0.841
Discouragement by Family	8	8	8	8	8	8	0.879
Online Presence	5	5	6	5	6	5	0.059
Workplace	6	7	7	6	7	6	0.073
Proximity	5	6	6	6	6	6.5	0.068
Availability	9	9	10	9	9	10	0.019
Appearance	8	8	9	8	9	7.5	0.033
Skincare	8	8	9	8	10	8	0.024
Cost	8	8	8	8	8	5.5	0.046
Professionalism	10	10	10	10	10	10	0.712

Demographic factors like religion, marital status, and nationality were consistently rated as least important across all governorates, each with median scores of 1.0-2.0, reaffirming their minimal influence in dermatologist choice. While most findings were not statistically significant, observable patterns suggest that residents of Mubarak Al-Kabeer and Ahmadi placed greater weight on cosmetic and professional presentation, whereas patients from Jahra valued experience and family influence slightly more. These regional differences, while subtle, could inform targeted communication and service tailoring by dermatologists serving different parts of Kuwait.

Effect of insurance coverage on dermatologist selection

**Figure 5:**
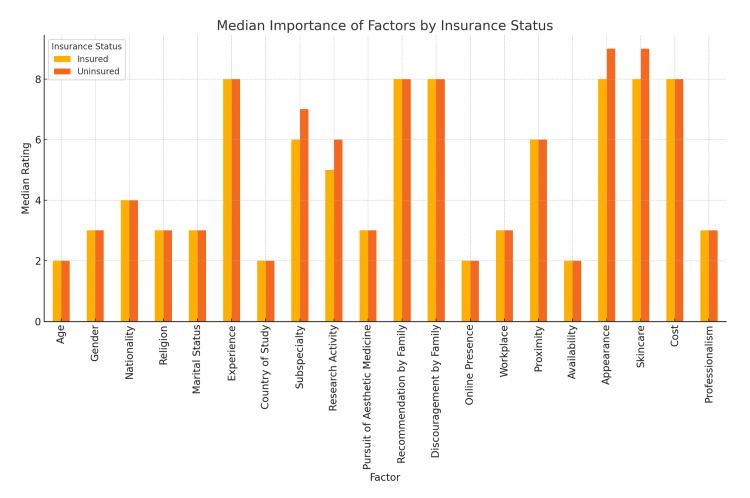
Comparison in Dermatologist Selection Between Insured and Non-Insured Participants

As of late, insurance coverage - and lack thereof - has become of growing importance in Kuwait, with many physicians perceiving it as a key factor in how patients choose their doctors (Figure 5). A comparison of dermatologist selection preferences between patients with and without health insurance revealed mostly similar trends across groups, though subtle differences appeared in certain domains, but had no statistical significance. Across nearly all variables, the median scores were consistent, indicating that insurance status did not dramatically shift core preferences. For instance, both insured and uninsured patients rated experience and professionalism highly, each with a median of 8.0 and 10.0, respectively. Likewise, availability and recommendation by family had a median of 9.0 and 8.0, respectively, in both groups. Nonetheless, slight differences were noted. The median rating for dermatologist's nationality was higher among insured patients (median=2) than the uninsured (median=1), indicating insured individuals may be more sensitive to cultural or regional identity. Similarly, subspecialty and research background were rated slightly higher among the uninsured (both median=7 and 6) versus insured (both median=6 and 5), possibly reflecting a desire among uninsured patients for greater reassurance in qualifications. Additionally, the personal skincare of a dermatologist was rated more important among the uninsured (median=9) than the insured (median=8), suggesting stronger attention to visual cues and credibility through personal appearance.

Demographic factors such as religion and marital status had low ratings (median=1.0) across both groups, reaffirming their minimal role in dermatologist selection, regardless of insurance coverage. Overall, while the presence or absence of insurance did not lead to statistically dramatic shifts, the observed differences in preference medians - particularly around academics and appearance - may be useful for tailoring services across patient types.

## Discussion

This study provides a comprehensive look into the multifaceted process by which patients in Kuwait select their dermatologists. It reveals that while various factors may be considered, patients consistently prioritize modifiable traits - particularly professionalism, availability, experience, and dermatologist's physical appearance - over fixed demographic attributes such as religion, nationality, or marital status (Table 4). These findings are reflected in existing literature, suggesting that physician behavior and perceived competence often overshadow non-modifiable characteristics when patients make care decisions [8,9].

**Table 4:** Top Five Patient Preference Factors Based on Actual Frequency (% Selection)

Factor	Most Selected Option	N	%
Professionalism vs. Friendliness	Professionalism	393	39.6
Availability	No waiting	289	29.1
Preferred Religion	Muslim	242	24.4
Aesthetic Pursuit	Highly favorable	219	22.1
Preferred Marital Status	Single	13	1.3

Professionalism was the highest-rated factor overall, with a median of 10 and a notably narrow interquartile range - with 10 being most important and 0 being not important at all. This emphasizes the weight patients place on physician conduct and demeanor, aligning with earlier findings that patients view professionalism as a key indicator of trustworthiness and a reflection of their potential quality of care [3,6]. Similarly, attributes such as experience, availability, and skincare were also rated highly - suggesting that both convenience and impression influence a patient’s decision-making.

Gender-based differences were particularly pronounced. Female participants consistently rated aesthetic and interpersonal factors higher than males, including personal appearance and skincare, reflecting a possibly greater emphasis on visual and empathetic cues. These results are in line with research indicating that women often place more value on relational dynamics and perceived competence in healthcare interactions [5,10].

Socioeconomic and regional comparisons added further depth to these observations. Higher-income individuals placed greater value on academic credentials, such as the country of study and research activity, echoing previous findings that more affluent patients may associate academic pedigree with clinical excellence [11]. Likewise, participants from certain governorates like Mubarak Al-Kabeer and Ahmadi prioritized physical presentation, whereas those in Jahra emphasized experience. These patterns suggest that dermatologists may benefit from tailoring their outreach strategies based on both the demographic profile of their patient population.

Interestingly, insurance coverage - despite being a growing concern within Kuwait’s healthcare landscape - did not markedly influence most preference variables, with the differences showing no statistical significance. However, subtle differences were observed: insured patients gave slightly higher importance to the dermatologist's nationality, while uninsured individuals placed greater emphasis on appearance, skincare, and academic indicators. These variations may reflect a compensatory mechanism among uninsured individuals to ensure quality through observable or reputational cues, a trend supported by previous findings in health access disparities [7,12].

Crucially, non-modifiable characteristics such as religion, marital status, and nationality were consistently rated the least important across all subgroups. Considering that most participants were Kuwaiti Muslims, it may be interesting to many that 61.5% had no preference on nationality and 75.6% had no preference on religion. This consistent trend aligns with studies showing that while such factors may shape patient comfort in specific contexts, they are largely overridden by clinical, professional, and interpersonal considerations in specialty care [4,13].

A comparison between preferences of those in Kuwait and those in countries such as the United States show both converging and diverging trends in physician selection. Both professionalism and experience remain the most important priorities for patients, reaffirming their significance in building trust and providing satisfaction. However, factors like a dermatologist’s appearance and skincare are less significant internationally as compared to in Kuwait, particularly among younger and female populations [9] . This may reflect the integration of aesthetic and cosmetic services into dermatological care, as well as sociocultural perceptions of physical appearance and personal presentation. Furthermore, fixed traits like gender, nationality, and religion had higher significance among those from other nationalities, but minimal significance among those in Kuwait (Table 5). These differences reflect the evolving values of patients in Kuwait and highlight the importance of care and results over demographic traits.

**Table 5:** Patient Preferences in Dermatologist Selection: Kuwait versus International Literature

Selection Factor	Findings in Kuwait (Current Study)	Typical Findings in International Studies
Professionalism	Highest rated factor (Median=10, IQR=2)	Consistently ranked among the top; key driver of trust and satisfaction
Availability/ Waiting Time	Very important (Median=9, IQR=3)	Often ranked top 3; ~75-85% of patients favor shorter wait times
Physical Appearance	High importance (Median=9); especially valued by females	Usually less emphasized; average scores ~5-7 where assessed
Clinical Experience	Highly valued (Median=8); highest in Jahra region	Considered important globally; often ranks among top selection criteria
Physician Gender	Low importance (Median=3); 55.4% had no gender preference	Mixed results; gender preference ranges from 25–40% in some regions
Nationality	Minimal impact (Median=2); 61.5% reported no preference	Often low relevance; most patients prioritize other traits
Religious Affiliation	Least important (Median=1); 75.6% had no preference	Rarely studied
Communication and Friendliness	54% valued both equally; 39.6% prioritized professionalism	Strong predictor of satisfaction; ranks highly in patient-centered models
Academic Background	44.5% preferred foreign-trained doctors; Research median=6	Valued more by educated or affluent groups; less consistent overall
Online Presence	67.4% viewed positively; 32.7% found it highly favorable	Emerging factor; increasingly relevant with rise of digital health
Physician’s Skincare (Personal)	Median = 8; Females=9 vs. Males=7	Rarely studied in international settings; culturally unique to aesthetic-focused care

One of the primary strengths of this study lies in its large and diverse sample size, which enabled meaningful comparisons across gender, income levels, and regional distribution. Moreover, the focus on both modifiable and non-modifiable traits offers a holistic view of patient preferences, bridging gaps seen in prior local literature. Nevertheless, certain limitations should be acknowledged. First, the survey instrument was not adapted by a validated questionnaire, but rather some questions were inspired to write our survey. Second, the questionnaire was not formally validated post-adaptation, which may impact the internal consistency and construct validity of certain items. Third, the reliance on self-reported data may introduce response bias or social desirability effects. Fourth, the sampling was a convenience sampling, and the online distribution of the survey may have led to underrepresentation of populations with limited internet access or reach, such as the elderly and certain governorates. Additionally, while the study captures preference, it does not assess actual behavior or satisfaction post-selection, which may differ. Finally, the study is cross-sectional in nature and cannot establish causality or observe changes over time.

Future research may benefit from incorporating qualitative methods, such as interviews or focus groups, to delve deeper into the reasons behind these preferences. Longitudinal studies could also explore how preferences shift over time or with different health experiences. As Kuwait’s healthcare sector continues to evolve, reevaluation of patient values will be essential to guide effective service delivery and subsequent success.

## Conclusions

The findings of this study affirm that when selecting dermatologists, patients in Kuwait predominantly prioritize modifiable traits - especially professionalism, experience, availability, and appearance - over intrinsic demographic factors. Gender, income, and regional variations do influence preferences to some extent, particularly regarding aesthetics and academic background. However, core values such as professionalism and experience remain universally important across all subgroups.

These insights are valuable for dermatologists and healthcare institutions aiming to improve patient satisfaction and engagement. Emphasizing timely availability, maintaining a professional and approachable manner, and clearly communicating credentials and expertise may significantly enhance patient trust and clinic utilization. As Kuwait’s healthcare environment continues to evolve, understanding patient expectations will be critical to shaping patient care.
